# Phase Transitions by an Abundant Protein in the Anammox Extracellular Matrix Mediate Cell-to-Cell Aggregation and Biofilm Formation

**DOI:** 10.1128/mBio.02052-20

**Published:** 2020-09-08

**Authors:** Thomas Seviour, Lan Li Wong, Yang Lu, Sudarsan Mugunthan, Qiaohui Yang, Uma Shankari, Irina Bessarab, David Liebl, Rohan B. H. Williams, Yingyu Law, Staffan Kjelleberg

**Affiliations:** a Singapore Centre for Environmental Life Sciences Engineering, Nanyang Technological University, Singapore; b A*STAR Microscopy Platform, Research Support Centre, Singapore; c Singapore Centre for Environmental Life Sciences Engineering, National University of Singapore, Singapore; d School of Biological Sciences, Nanyang Technological University, Singapore; e School of Biological, Earth and Environmental Sciences, University of New South Wales, Sydney, Australia; Korea Advanced Institute of Science and Technology

**Keywords:** anammox, aggregation, biofilm, droplets, extracellular matrix, phase transitions, surface proteins

## Abstract

This study describes the first direct functional assignment of a highly abundant extracellular protein from a key environmental and biotechnological biofilm performing an anaerobic ammonium oxidation (anammox) process. Expression levels of Brosi_A1236, belonging to a class of proteins previously suggested to be cell surface associated, were in the top one percentile of all genes in the “*Candidatus* Brocadia sinica”-enriched biofilm. The Brosi_A1236 structure was computationally predicted to consist of immunoglobulin-like anti-parallel β-strands, and circular dichroism conducted on the isolated surface protein indicated that β-strands are the dominant higher-order structure. The isolated protein was stained positively by the β-sheet-specific stain thioflavin T, along with cell surface- and matrix-associated regions of the biofilm. The surface protein has a large unstructured content, including two highly disordered domains at its C terminus. The disordered domains bound to the substratum and thereby facilitated the adhesion of negatively charged latex microspheres, which were used as a proxy for cells. The disordered domains and isolated whole surface protein also underwent liquid-liquid phase separation to form liquid droplets in suspension. Liquid droplets of disordered protein wet the surfaces of microspheres and bacterial cells and facilitated their coalescence. Furthermore, the surface layer protein formed gels as well as ordered crystalline structures. These observations suggest that biophysical remodeling through phase transitions promotes aggregation and biofilm formation.

## Abstract (precis)

By employing biophysical and liquid-liquid phase separation concepts, this study revealed how a highly abundant extracellular protein enhances the key environmental and industrial bioprocess of anaerobic ammonium oxidation (anammox). Extracellular proteins of environmental biofilms are understudied and poorly annotated in public databases. Understanding the function of extracellular proteins is also increasingly important for improving bioprocesses and resource recovery. Here, protein functions were assessed based on theoretical predictions of intrinsically disordered domains, known to promote adhesion and liquid-liquid phase separation, and available surface layer protein properties. A model is thus proposed to explain how the protein promotes aggregation and biofilm formation by extracellular matrix remodeling and phase transitions. This work provides a strong foundation for functional investigations of extracellular proteins involved in biofilm development.

## INTRODUCTION

There is an increasing awareness that biofilm biology is determined by extracellular polymers that imbue biofilm matrices with their emergent properties, such as increased stress tolerance (1). In contrast to, for example, housekeeping or metabolic functions in cells, there are no classifications or standard descriptions of extracellular functions, which reflects the diversity of function but also the complexity of many biofilm systems. There are also experimental hurdles to extraction, isolation, and structural elucidation of matrix exopolymers that confound attempts to resolve their functions. Hence, there is a need for functional characterization of extracellular polymers (2), as well as the development of model exopolymers for generating a database of functional descriptions.

Anaerobic ammonium oxidation (anammox) is the direct oxidation of ammonium by nitrite to nitrogen (3). This achieves nitrogen removal from wastewater with a lower aeration requirement than conventional processes (i.e., nitrification-denitrification). Anammox is a major contributor to nitrogen losses from marine environments (4) and is carried out by bacteria of the phylum *Planctomycetes* which are further classified into five known genera (“*Candidatus* Kuenenia,” “*Candidatus* Brocadia,” “*Candidatus* Scalindua,” “*Candidatus* Jettenia,” and “*Candidatus* Anammoxoglobus”) (5). Furthermore, anammox predominately takes place in community biofilms, as it depends on mutualistic associations with other N-cycling organisms, such as nitrifiers (6, 7). The anammox process is prevalent across marine, freshwater, and terrestrial ecosystems (5), is of significant industrial interest (i.e., wastewater remediation), and relies on bacteria living in biofilm communities embedded in an extracellular matrix. It hence represents a key model for understanding biofilm exopolymer structure-function.

Proteins have recently been suggested to play a structural role in anammox community extracellular polymeric substances (EPS) (8), particularly cross β-sheet proteins, O-glycosylated glycoproteins (9) and possibly even amyloids (10). Nonetheless, no direct functional characterization has been undertaken on a known or isolated anammox biofilm extracellular protein. In the absence of characterized reference extracellular proteins for important industrial and environmental anammox biofilms (11), direct functional characterization on isolated exoproteins is the only strategy available to explain how they mediate anammox biofilm structure and function.

Progress in identifying extracellular protein targets in environmental systems such as anammox biofilms is limited compared to that for single-species model biofilm systems (e.g., Pseudomonas aeruginosa and Bacillus subtilis), which likely reflects the fact that anammox bacteria are unavailable in pure culture and genetically intractable (11). This makes applying classical microbiological approaches to functional assignment challenging. Accordingly, anammox biofilm extracellular proteins remain poorly annotated in public databases.

To address this shortcoming, we previously developed a method involving a novel solvent (i.e., ionic liquid 1-ethyl-3-methylimidazolium acetate [EMIM-Ac]) for extracting and isolating an extracellular protein from “*Candidatus* Brocadia sinica”-enriched anammox biofilms (12). We identified the glycoprotein Brosi_A1236 as the most abundant protein in the “*Ca*. Brocadia sinica”-enriched biofilm extracellular extract (12), which belongs to the class of glycoproteins extracted from other anammox sludges, including Kustd1514 from “*Candidatus* Kuenenia stuttgartiensis”-enriched sludge and its homolog from “*Candidatus* Brocadia sapporoensis”-enriched sludge (9, 13). These are a class of anammox proteins similar to the heme C proteins that perform common reactions in anammox metabolism (e.g., hydrazine synthase). They have also been suggested to appear on the surfaces of cells and are therefore extracellular. Despite being highly expressed, they remain poorly characterized and, as general anammox proteins, are strong candidates for elucidating the role of extracellular proteins in anammox biofilms and unravelling the emergent properties of community and industrial biofilms more broadly.

Here, we undertook a functional and structural analysis on the isolated putative surface layer (S-layer) protein Brosi_A1236 and its key domains, to address the hypothesis that the glycoprotein plays an important structural role in anammox biofilms. We assessed the protein’s morphology and function by biophysical methods and investigated the possibility that Brosi_A1236 has β-sheet protein structures, as has been observed for exopolymers in the matrix of anammox biofilms (e.g., see reference 8). We further show that the C terminus of the surface protein contains two repeat regions of approximately 100 amino acids that are intrinsically disordered. Low-complexity or intrinsically disordered domains (IDDs), which are regions of the protein without three-dimensional order, have been implicated in biologically regulated phase separation of macromolecules into liquid-phase condensates (14). Phase separation occurs due to their tendency toward weak and transient interactions. The result is adhesive droplets with fluid properties suspended in aqueous solutions (i.e., liquid-liquid phase separation [LLPS]) (15). LLPS often leads to formation of non-membrane-bound organelles and is increasingly recognized as a way for eukaryotic cells to achieve intracellular compartmentalization, where droplets exist initially as metastable liquid condensates but can subsequently develop into gels and even solids (16). Furthermore, IDDs can make otherwise structured proteins form adhesive liquid droplets in solution (17).

Given that biofilms can be considered biopolymeric gels (18), we sought to explore the hypothesis that the surface protein Brosi_A1236 can promote biofilms by forming an adhesive, phase-separated liquid layer that initially wets cell surfaces and that can subsequently contribute to the bulk extracellular matrix as a gel. Elucidating the functions of a highly expressed and common anammox biofilm extracellular protein could additionally inform the environmental conditions that influence their expression and thus lead to improved strategies for managing anammox biofilm formation under different environmental conditions.

## RESULTS

### “*Ca*. Brocadia sinica” S-layer protein is highly expressed and has a large amount of β-sheet structures.

**FIG 1:**
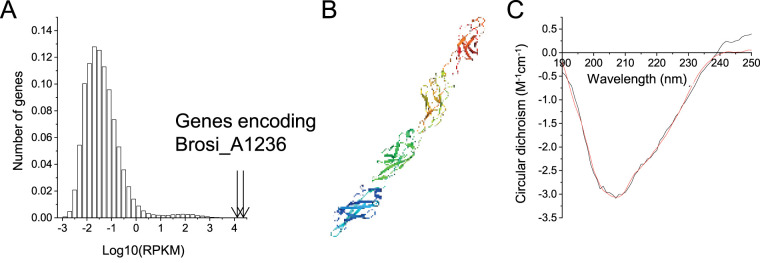
(A) Histogram of mean expression levels (log_10_ reads for kilobase of transcript per million mapped reads [RPKM]) of 404,234 genes in the “*Candidatus* Brocadia sinica”-enriched bioreactor community. Mean RPKM values were calculated across 11 samples taken throughout a typical anammox operating cycle. Arrows show the expression levels of gene fragments annotated to Brosi_A1236. Each bar represents the fraction of genes (*y* axis) with a log_10_ expression (in RPKM) that falls within the ranges represented by the *x* axis. (B) Structure predicted by I-TASSER of putative surface protein Brosi_A1236 amino acids 1141 to 1563, consisting of four immunoglobulin-like folds in β-sandwich conformation. (C) Experimental (black) and fitted (i.e., BestSel structure prediction server, red) circular dichroism spectrum of Brosi_A1236 isolated from “*Ca*. Brocadia sinica”-enriched biofilm in double-distilled water.

Average transcription levels, across a full cycle study, of the gene encoding putative surface protein Brosi_A1236 (GenBank accession no. GAN32721) were high, relative to all genes in the community (Fig. 1A). The protein structure was estimated by computational methods (19) to consist of approximately 50% β-sheet and 50% loop, with a transmembrane helix domain from amino acids 27 to 44. The structures of truncated amino acid fragments were additionally predicted by I-TASSER (20) (amino acids 1 to 400, 401 to 800, 801 to 1200, and 1141 to 1563) to form fibers, consisting of immunoglobulin-like domains of approximately 100 amino acids in an anti-parallel β-strand configuration (Fig. 1B). Finally, the circular dichroism (CD) spectrum of the isolated fraction (Fig. 1C) was consistent with that described by Lotti et al. (8) for their anammox biofilm extracellular matrix, with a trough at 207 to 208 nm and a gradual increase in CD toward normal at 240 nm. This was subsequently interpreted to be due to 26.7% β-sheet, 16.6% α-helix, 12.7% β-turn, and 44% random coil protein structures (21).

### Isolated surface protein stained by thioflavin T.

**FIG 2:**
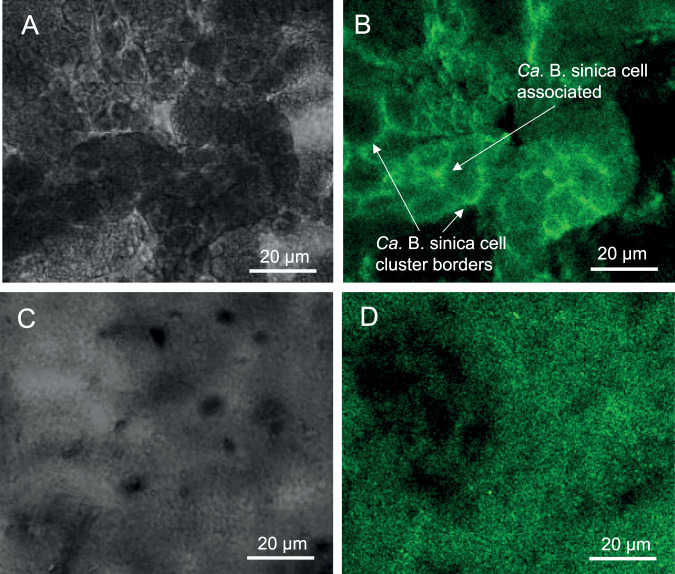
(A) Confocal and (B) bright-field micrographs of “*Candidatus* Brocadia sinica”-enriched anammox biofilm stained with thioflavin T (0.5% [wt/vol]). (C) Confocal and (D) brightfield micrographs of isolated S-layer protein Brosi_A1236 stained with thioflavin T (0.5% [wt/vol]). Bar, 20 μm.

Thioflavin T (ThT) is used for amyloid fibril detection due to its preference for binding to β-sheet protein structures (22). Two distinct regions of “*Ca*. Brocadia sinica”-enriched biofilm were stained by ThT; one in which the ThT stain appears, based on fluorescent *in situ* hybridization images published by Wong et al. (12), to be associated with “*Ca*. Brocadia sinica” cell clusters (i.e., putatively cell-associated) and the other at the borders of the “*Ca*. Brocadia sinica” cell clusters (Fig. 2A and B). The putative S-layer protein Brosi_A1236 also binds strongly to ThT (Fig. 2C and D). This is in contrast to the ThT-stained image of a biofilm of Pseudomonas aeruginosa PAO1 (see Text S1 and Fig. S1 in the supplemental material), known to produce only low levels of extracellular β-sheet protein (23), which shows no overlap of ThT with the extracellular protein-specific Sypro Ruby biofilm matrix stain in the matrix of the P. aeruginosa biofilm, illustrating that ThT binding is not a general property of all biofilm matrices.

10.1128/mBio.02052-20.6 TEXT S1 Supplemental materials and methods: Pseudomonas aeruginosa biofilm growth assay (i.e., ThT control). Download Text S1, DOCX file, 0.02 MB. Copyright © 2020 Seviour et al. 2020 Seviour et al. This content is distributed under the terms of the Creative Commons Attribution 4.0 International license.

10.1128/mBio.02052-20.1 FIG S1 (A) Confocal (green: thioflavin T, 0.5% [wt/vol]; red: FilmTracer SYPRO Ruby biofilm matrix stain). (B) Bright-field and (C) overlapping micrographs of a 4-day-old Pseudomonas aeruginosa (PAO1) biofilm. These show that coincident binding to thioflavin T and Ruby takes place within cells only (yellow) and not within extracellular matrix and that only the extracellular region is stained by Ruby biofilm matrix stain (red regions between cells). Bar, 10 μm. Download FIG S1, PDF file, 0.4 MB. Copyright © 2020 Seviour et al. 2020 Seviour et al. This content is distributed under the terms of the Creative Commons Attribution 4.0 International license.

### A disordered C terminus makes the anammox biofilm S-layer protein adhesive.

Further analysis of the structure of the anammox biofilm S-layer protein revealed that its C terminus has two repeat domains with 84% similarity, from amino acids 1254 to 1338 and 1354 to 1439 (Fig. S2A). There is also a high degree of intrinsic disorder from amino acid 1228 to the C terminus, which spans these repeat regions (Fig. S2B). IDDs are typically enriched in the polar amino acids threonine, serine, and glycine, which are observed here for the anammox biofilm surface protein (i.e., 16.0, 11.8, and 11.4%, respectively). This enrichment promotes conformational heterogeneity that renders them effective molecular adhesins (16, 17).

10.1128/mBio.02052-20.2 FIG S2 (A) C-terminal repeat domains of Brosi_A1236 from amino acids 1254 to 1338 (i) and 1354 to 1439 (ii). (B) Intrinsic disorder prediction for Brosi_A1236 by Predictor of Natural Disordered Regions (PONDR), with a disorder prediction score of >0.5 indicating disordered regions. Download FIG S2, PDF file, 0.3 MB. Copyright © 2020 Seviour et al. 2020 Seviour et al. This content is distributed under the terms of the Creative Commons Attribution 4.0 International license.

Therefore, to investigate the possibility that the disordered C terminus might contribute to and help explain the role of the surface protein in the extracellular matrix of a “*Ca*. Brocadia sinica”-enriched biofilm, we produced protein constructs from its intrinsically disordered region, spanning each of the repeat regions (i.e., amino acids 1254 to 1338 and 1354 to 1439) and the C-terminal domain (1448 to 1551) (Fig. S3). We then incubated carboxylated fluorescent microspheres of different colors (red or green) with various combinations of the constructs to determine whether the intrinsically disordered C terminus could mediate protein adhesion.

10.1128/mBio.02052-20.3 FIG S3 Coomassie blue stain of an SDS-PAGE gel of anammox biofilm surface protein construct repeat domain 1 (i.e., amino acids 1254 to 1338) (A) and repeat domain 2 (i.e., amino acids 1354 to 1439) (B) recombinantly expressed with Escherichia coli. Download FIG S3, PDF file, 0.2 MB. Copyright © 2020 Seviour et al. 2020 Seviour et al. This content is distributed under the terms of the Creative Commons Attribution 4.0 International license.

**FIG 3:**
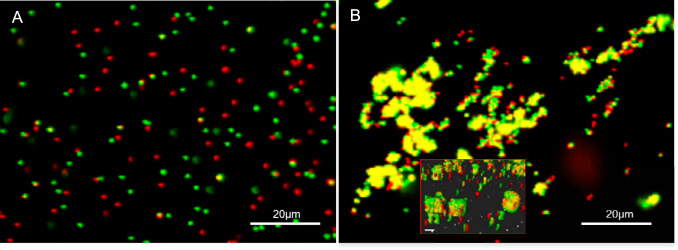
(A) Confocal scanning laser microscope image of protein binding assay showing the dispersal of green and red microspheres following preincubation with a nonbinding protein (i.e., bovine serum albumin [negative control]). Note that the majority of beads with different fluorescence are observed separately (i.e., as red or green only), with a few overlapping signals (i.e., yellow) due to random positional overlay. (B) Coalescence of green microspheres incubated with construct 1 (i.e., anammox biofilm S-layer protein amino acids 1254 to 1338) and red microspheres incubated with construct 2 (i.e., amino acids 1354 to 1439). (Inset) Three-dimensional side view of coalesced beads, demonstrating that binding derives from protein interactions and not the overlapping of beads. The samples were prepared in 50 mM MES–5 mM CaCl_2_, pH 6.0.

As illustrated by the negative control, the green and red beads remain clearly dispersed following preincubation with a nonbinding protein (i.e., bovine serum albumin [BSA]) (Fig. 3A). The green and red beads coalesced to form yellow clusters when preincubated with a protein known to complex with itself (i.e., cadherin in the presence of calcium) (Fig. S4). As with the positive control, all combinations of red and green microspheres gave a positive binding response (i.e., yellow clusters) when preincubated with any pair of our protein constructs, as illustrated for the constructs of surface protein Brosi_A1236 from amino acids 1254 to 1338 and 1254 to 1439 (Fig. 3B). Thus, the intrinsically disordered domains of the anammox biofilm surface protein bind strongly to each other.

10.1128/mBio.02052-20.4 FIG S4 Protein binding assay showing the coalescence of green and red microspheres with both preincubated with cadherin in the presence of 20 mM calcium (i.e., positive control). Download FIG S4, PDF file, 0.2 MB. Copyright © 2020 Seviour et al. 2020 Seviour et al. This content is distributed under the terms of the Creative Commons Attribution 4.0 International license.

### Brosi_A1236 and its intrinsically disordered region undergo phase condensation to liquid droplets.

**FIG 4:**
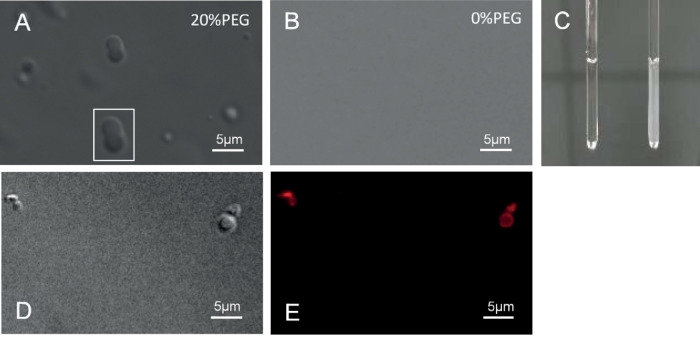
Bright-field micrograph of an anammox biofilm surface protein construct (amino acids 1354 to 1439) in an aqueous solution of 20 mM Tris (pH 7.5)–125 mM NaCl–2 mM DTT, 4°C, with (A) and without (B) polyethylene glycol at 100 μM), showing that liquid droplet formation in the Tris solution is induced by PEG. (C) Photograph of solution of an anammox biofilm surface protein construct (amino acids 1254 to 1338) in a 4-mm glass tube in the absence (left) and presence (right) of 20% (wt/vol) PEG, showing increased turbidity following PEG addition (C). (D and E) Bright-field (D) and epifluorescence (E) micrographs of isolated anammox biofilm surface protein (0.6 μM) in an aqueous solution of 20 mM Tris (pH 7.5)–125 mM NaCl–2 mM DTT, 4°C, labeled with protein-specific Alexa Fluor 647 dye (Thermo Fisher). This shows that the liquid droplets formed in the Tris solution are proteinaceous.

Phase condensation was induced in the intrinsically disordered region of the anammox biofilm surface protein (i.e., amino acids 1254 to 1338), yielding spherical liquid droplets from 0.5 to 2 μm upon addition of the crowding agent polyethylene glycol (PEG) (Fig. 4A). In contrast, such droplets did not result in the solutions without PEG (Fig. 4B) or in those with PEG but no protein construct (Fig. S5). Further evidence of the ability of the surface protein to undergo liquid-liquid phase separation was provided in terms of increased solution turbidity (Fig. 4C) and images of smaller liquid droplets fusing to create larger droplets (Fig. 4A, white box). Liquid-liquid phase condensation was also observed in solutions of the whole surface protein, even in the absence of PEG, in terms of the appearance of 3-μm droplets that were confirmed to be protein by Alexa Fluor 647 staining (Fig. 4D and E).

10.1128/mBio.02052-20.5 FIG S5 Bright-field micrograph of an aqueous solution of 20 mM Tris (pH 7.5)–125 mM NaCl–2 mM DTT, 4°C, following the addition of PEG (20% [wt/vol]). Download FIG S5, PDF file, 0.01 MB. Copyright © 2020 Seviour et al. 2020 Seviour et al. This content is distributed under the terms of the Creative Commons Attribution 4.0 International license.

### Liquid condensates can wet cell surfaces, mediate cell attachment, and coalescence.

**FIG 5:**
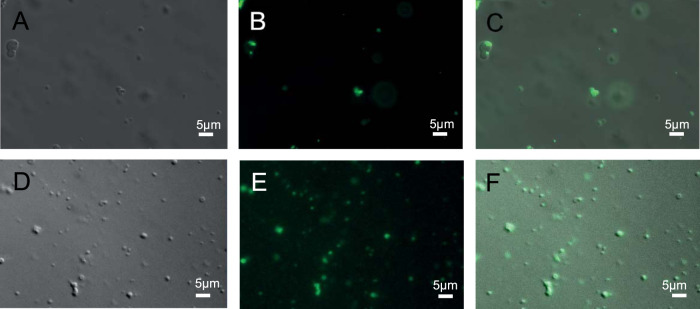
Bright-field (A and D), epifluorescence (B and E), and superimposed (C and F) micrographs of liquid droplets of an anammox biofilm surface protein construct (amino acids 1254 to 1338) (100 μM) in an aqueous solution of 20 mM Tris (pH 7.5)–125 mM NaCl–2 mM DTT, 4°C, and polyethylene glycol (20% [wt/vol]) following the addition of 0.5-μm carboxylate-modified latex microspheres (green) (B and C) and green fluorescence protein-labeled Pseudomonas aeruginosa cells (E and F) showing the wetting and protein droplet-mediated coalescence of microspheres and bacterial cells, respectively.

Carboxylate-modified latex microspheres (0.5 μm) with cell-like surface properties (24) were added to a solution containing liquid droplets of anammox biofilm surface protein amino acids 1254 to 1338 (Fig. 5A). Microspheres accumulated within the liquid droplets in preference to remaining suspended in water, as indicated by the intensity of the fluorescence signal emitted from the droplets after the microspheres were added (Fig. 5C). Multiple latex microspheres were found inside the droplets, which wet and provide a medium for aggregation of the latex microspheres.

This was repeated for green fluorescent protein-labeled Pseudomonas aeruginosa PAO1 cells introduced to constructs of the intrinsically disordered domains of an anammox biofilm surface protein that had undergone liquid-liquid phase separation (Fig. 5D). As with the microspheres, P. aeruginosa cells entered the droplets and appeared to fluoresce with greater intensity, indicative of a higher concentration within the cells (Fig. 5D), although many cells remained motile in the presence of liquid droplets of intrinsically disordered domains from anammox biofilm surface protein.

### Anammox biofilm surface protein forms gels and possibly latticed crystals.

**FIG 6:**
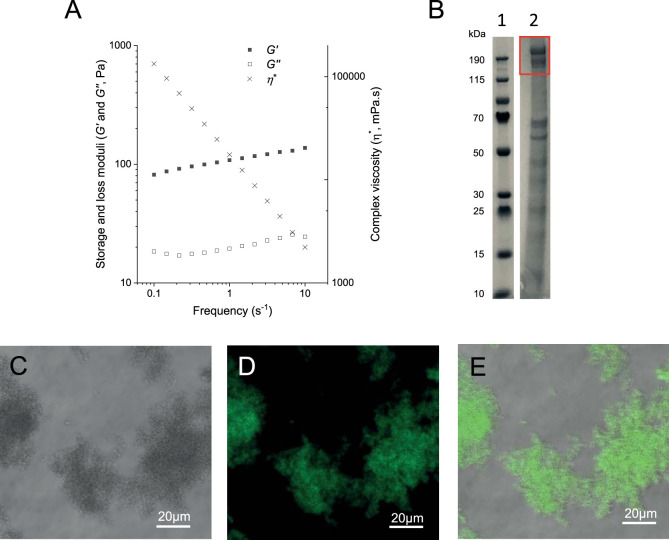
Gel of enriched extract of anammox biofilm surface protein dialyzed against double-distilled water (MWCO, 3,500 Da). Results are presented in terms of its rheological behavior described as gel or storage (*G*′) and viscous or loss (*G*ʺ) moduli, as well as complex viscosity (η*) (A) and in terms of protein size distribution in an SDS-PAGE gel (lane 1, ladder; lane 2, extract; the red rectangle highlights Brosi_A1236) (B). Bright-field (C), fluorescence (D), and superimposed (E) micrographs stained with thioflavin T (0.5% [wt/vol]) are also shown.

The “*Ca*. Brocadia sinica”-enriched anammox biofilm matrix is enriched in the S-layer protein Brosi_A1236, which forms a viscoelastic gel when dialyzed against water (12). This gel property is illustrated by a gel or storage modulus (*G*′) that is greater than the viscous or loss modulus (*G*ʺ) along the full frequency range (Fig. 6A). The gel-forming constituent is enriched in the anammox biofilm surface protein (SDS-PAGE gel run as described in reference 12 [Fig. 6B]). The isolated surface protein did not form a gel under the same conditions (dialysis [3-kDa molecular weight cutoff] against double-distilled water at 4°C). This suggests that either sample preparation was not optimized for gelation or it relies on interaction with other proteins to form physical cross-links. As for the isolated surface protein, the surface protein-enriched gel-forming extract stained positive for ThT (Fig. 6C to E). This further implicates the putative surface layer proteins of anammox biofilm as the β-sheet gel-forming extracellular constituent described in other systems (10).

**FIG 7:**
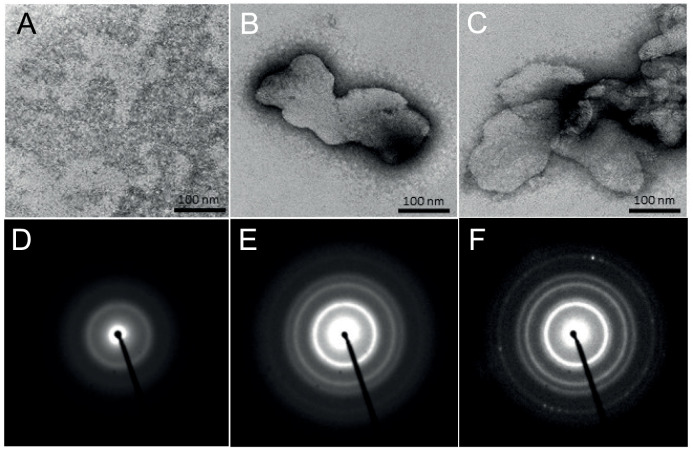
Transmission electron microscopy images of anammox biofilm surface protein (0.1 mg/ml), contrasted by negative staining, without preincubation with CaCl_2_ (A) and following incubation with 10 mM CaCl_2_, generating sheet-like structures (B and C). Selected area diffraction images of anammox biofilm surface protein without (D) and with (E and F) preincubation with 10 mM CaCl_2_ showing patterns characteristic of amorphous and polycrystalline structures, respectively.

To assess whether purified surface protein can assemble into the higher-order paracrystalline structures that are characteristic of surface proteins, we performed ultrastructural analysis by transmission electron microscopy (TEM) of proteins deposited onto carbon-coated grids. In the absence of calcium, no higher-order structures were detected (Fig. 7A). However, when protein was incubated with 10 mM calcium, which is one of the major ions in anammox biofilms (25), large sheet-like structures were formed on the grid surface (Fig. 7B and C). Although the morphology of the lattice (i.e., periodicity) could not be discerned from the TEM images, the electron diffraction pattern showed that the continuous diffraction rings that are characteristic of polycrystalline lattices and consistent with S-layer proteins were generated when the surface protein was preincubated in the presence of calcium (Fig. 7E and F), in contrast to samples not preincubated with calcium (Fig. 7D).

## DISCUSSION

### A model protein for describing the role of an extracellular protein in anammox biofilms.

Building up structural and functional descriptions of extracellular proteins in public databases, particularly in environmental and industrial biofilms, will inform on biofilm-growth regulation and control, enhance the operation and design of biofilm-based bioprocesses (e.g., wastewater treatment), and potentially advance the water and wastewater sectors toward improved circular economy and resource recovery (2). One potential bottleneck in achieving this is the lack of consensus model exoproteins for focused structure-function characterization studies. Here, we employed the surface protein Brosi_A1236 from “*Ca*. Brocadia sinica”-enriched systems as a consensus exoprotein for anammox biofilms. Although the method applied does not solubilize the entire biofilm, we show from transcription levels that Brosi_A1236 was among the most highly expressed proteins in the “*Ca*. Brocadia sinica”-enriched anammox biofilm. Moreover, this was consistent with high levels of this protein found in our earlier study for the biofilm and its extracellular matrix (12), and we here provide direct biophysical evidence that the anammox surface protein has structural functions.

Furthermore, we show that “*Ca*. Brocadia sinica” surface protein homologues contribute to the extracellular matrix of other anammox biofilms (i.e., in addition to putative S-layer protein Kustd1514 described by van Teeseling et al. [13]). These homologues are detected by the β-sheet protein-specific ThT stain, as described for the anammox biofilm EPS by Lotti et al. (8). In our study, the extracellular regions of the “*Ca*. Brocadia sinica”-enriched biofilm as well as the surface protein isolate stained positively with ThT. EPS from the anammox biofilms does not contain cell membrane components (specifically phospholipids or lipopolysaccharides) (12), demonstrating that cell membranes are not fluidized during extraction. Thus, the binding of ThT to extracted extracellular protein cannot be explained by contamination by cellular components. Additionally, the circular dichroism of our surface protein isolate displayed the structural features described by Lotti et al. (8) for the EPS from their anammox system, with computational predictions indicating a high fraction of β-structures. It is thus possible that the β-sheet structures described by Lotti et al. (8), which they suggested were responsible for amyloid fibrils observed in their biofilm, are from the same class of proteins as Brosi_A1236. This further establishes the credentials of the anammox biofilm surface protein described in this study as a surface protein of broader interest.

### Anammox biofilm surface protein is unlikely to form β-sheet fibrils in the matrix.

We sought to elucidate the role of the anammox biofilm surface protein based on properties inferred from its composition and structure. We aimed to reconcile its potential functions in promoting either (i) cell-cell adhesion (i.e., through cell wetting or directly mediating contacts) or (ii) the formation of amyloid fibrils, which were shown by Lotti et al. (10) to be structural agents in their anammox biofilms. We specifically considered the effects of domains of intrinsically disordered structures that are known to promote adhesion and liquid-liquid phase separation even in otherwise structured proteins (17). We observed that microspheres incubated with constructs composed of IDDs bound to each other, which supports our hypothesis that they are adhesive, a property required for cell surface proteins promoting cell-cell surface attachment. Amyloid fibrillation can also proceed through the end-to-end attachment of ordered β-sheet fibrils via sticky or “fuzzy” C-terminal IDD (26). Regions of intrinsic disorder therefore do not preclude the possibility that the protein also forms fibrillar structures. Additionally, two distinct regions stained positively for ThT in the biofilm (Fig. 2B), which could indicate either that different β-sheet proteins were present or that the same β-sheet protein appeared in different regions in the biofilm, i.e., as cell surface and matrix proteins, possibly even with two different morphologies (including β-sheet fibrils). As described by Wong et al. (12), SDS-PAGE analysis of the purified anammox biofilm surface proteins indicated that two proteins of 170 and 200 kDa were isolated. Both proteins yielded a positive immunoblot when exposed to the Brosi_A1236 antibody, and it is therefore likely that they are Brosi_A1236 at different stages of processing (e.g., before and after cleavage of the membrane-bound domain). Different proteoforms could explain why different regions of the biofilm stain positively with ThT.

Based on other behaviors described in this study, however, it is unlikely that the surface protein forms β-sheet fibrillated structures at either location in the biofilm. We observed that the isolated surface protein (Fig. 2D) formed a dense, predominately amorphous film and not fibrillated structures. While failure of the protein to fibrillate could be attributed to sample preparation not optimized for fibril maturation (27), we also observed LLPS for IDD constructs (Fig. 4A) and the whole surface protein isolate (Fig. 4D). While IDD can promote amyloid fibril elongation (26), to the best of our knowledge, in doing so they do not disrupt the ordered β-sheet fibril core. Here, the complete anammox biofilm surface protein underwent LLPS. The whole protein therefore became disordered. Such behavior is inconsistent with proteins forming amyloid fibrils.

### Liquid-phase condensates can promote direct and indirect cell coalescence.

We observed here the partitioning of negatively charged microspheres (i.e., as surrogates for cells) and nonmotile bacterial cells into liquid-phase condensates. The cells were coated by concentrated liquid droplets of the anammox biofilm surface protein IDD. Given the adhesive properties of the intrinsically disordered region, this condensate forms an adhesive wetting layer on the surface of anammox cells in aqueous environments that could facilitate direct cell-cell attachment. Alternatively, the protein droplets of the intrinsically disordered domain of anammox biofilm surface proteins could also provide a medium within which cells could coalesce through space (i.e., without direct cell-cell contact).

We also identified two key behaviors of the surface protein. First, in an intermediate stage of isolation, it contributes to gel formation. Liquid-phase condensates can develop into gels. Thus, allowing for long-range (i.e., through matrix or droplets) coalescence of cells or cell clusters, liquid-liquid phase condensates of the surface protein could subsequently develop gel-like properties, which is one of the key biophysical traits of biofilms (28). Second, the protein has the capacity to assemble into polycrystalline sheet-like (i.e., S-layer like) structures either on inert surfaces or at the air-liquid or liquid-solid interface, as evidenced by continuous diffraction rings in the electron diffraction pattern for protein preincubated with calcium (Fig. 7D). This result, coupled with observations of liquid condensation, indicates that the surface protein may proceed from an adhesive cell-wetting protein to a paracrystalline state (i.e., a more fixed state following direct cell-cell adhesion). Further analysis, however, is necessary to fully elucidate the conditions contributing to phase transition of the anammox biofilm surface protein (i.e., to paracrystalline lattice). Furthermore, it is possible that the surface protein is structurally promiscuous and can form condensed states with different material properties, including gels, droplets, and crystals (29).

### Proposed mechanism for role of the surface protein in promoting anammox biofilms.

**FIG 8:**
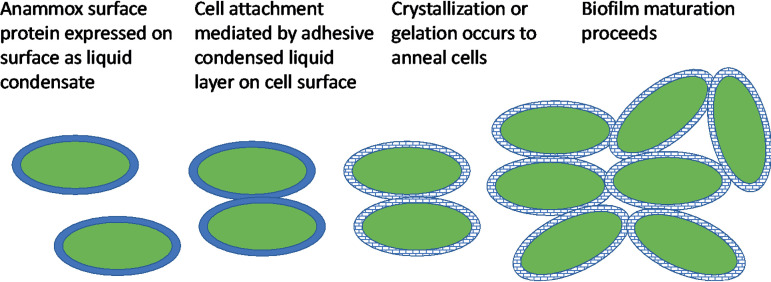
Schematic describing the role of the adhesive wetting layer of the anammox biofilm surface protein in potentially promoting anammox bacteria cell-cell attachments and nucleating biofilm development.

Our finding that the ThT-positive stain is localized at the surface of the cells (Fig. 2A) is consistent with the work of van Teeseling et al. (13), who described the appearance of an anammox biofilm surface layer protein (Brosi_A1236 homologue Kustd1514 in “*Candidatus* Kuenenia stuttgartiensis”-enriched cultures) at the surface of and junction between anammox cells. While immunofluorescence microscopy using antibodies raised against the anammox biofilm surface protein is required to confirm that the ThT signal in both regions of the biofilm (Fig. 2A) is due to the surface protein, its ability to exist in different forms suggests that it could contribute structurally to the anammox biofilm across both domains by two different mechanisms. First, in the region where ThT is restricted to the cell surfaces, the ability of the surface protein to form adhesive wetting layers would allow it to facilitate initial cell-cell attachment followed by crystallization to aggregate the cells. This is analogous to the description of S-layer protein crystallization as proceeding via a liquid phase (30) (see Fig. 8 for a schematic). Second, in the ThT-positive amorphous matrix region, the surface protein may contribute to anammox biofilm structure as a gel (28).

The constructs of the intrinsically disordered regions of anammox biofilm surface protein (e.g., amino acids 1254 to 1338) were expressed in unglycosylated form. Given the tendency of glycosylation to target intrinsically disordered regions, it is possible that in the surface protein these regions are glycosylated. Nonetheless, glycosylation of intrinsically disordered regions occurs due to the need for structural pliability associated with conformational changes (31). The behavior of nonglycosylated amino acids in this region represents one end of the conformational spectrum, and the idea of posttranslational modifications and structural versatility is in agreement with the model of an anammox biofilm surface protein playing several structural roles in anammox biofilms.

To confirm the model in which the anammox biofilm surface protein plays different roles across different locations due to its ability to exist in different condensed forms, however, a more detailed analysis of its phase transition behavior under biofilm-relevant conditions (e.g., highly crowded with osmotic gradients) is required, principally with regard to the transitions from solution to droplet, solution to gel, solution to crystal, droplet to crystal, and droplet to gel. Such an analysis would inform the choice of environmental conditions to manage phase transitions and ultimately control anammox biofilm structure to deliver optimized anammox bioprocesses.

## MATERIALS AND METHODS

### Extracellular-protein extraction.

“*Candidatus* Brocadia sinica”-enriched anammox biofilm was collected from a laboratory-scale sequencing batch reactor, seeded with activated sludge from a full-scale used-water reclamation plant in Singapore. The reactor was fed synthetic medium containing (grams per liter) (NH_4_)_2_SO_4_ (2.12), NaNO_2_ (1.48), KHCO_3_ (1.25), KH_2_PO_4_ (0.025), CaCl_2_ · 6H_2_O (0.3), MgSO_4_ · 7H_2_O (0.2), and FeSO_4_ · 7H_2_O (0.025), as well as 1.25 ml/liter of trace mineral solution as described by de Graaff et al. (32). Argon-CO_2_ was sparged continuously at 30 ml/min throughout the cycle to prevent ingress of oxygen. A heating jacket was used to maintain the temperature at 35 ± 0.05°C. Further information on reactor operation and microbial community analysis is provided in reference 33. The collected biofilms were washed with Milli-Q water and lyophilized. Freeze-dried anammox biofilm was added to 15 ml of a 40% (vol/vol) EMIM-Ac–60% *N*,*N*-dimethylacetamide (DMAc) solution to a final concentration of 30 mg biofilm/ml (34). The biofilm was incubated in the EMIM-Ac–DMAc at 55°C for 16 h. The soluble fraction was collected, and ethanol was added (70% [vol/vol]). The precipitate was collected by centrifugation (10,000 × *g*), dialyzed against double-distilled water for 48 h (SnakeSkin dialysis tubing; 3,500 molecular weight cutoff [MWCO]; 22 mm), and lyophilized (FreeZone Plus 4.5-liter cascade benchtop freeze-dry system).

### Purification of anammox biofilm extracellular protein.

Crude lyophilized protein extract was dissolved in 20 mM HEPES, pH 8.0, passed through a 20-μm filter, and then purified in an anion-exchange column connected to an Amersham Äkta fast protein liquid chromatograph (FPLC). The EPS mixture was loaded onto a Source15Q column pre-equilibrated with HEPES and eluted with a linear salt gradient of 500 mM NaCl. Fractions containing Brosi_A1236 were combined, concentrated with a rotary evaporator, and stored at −20°C for further analysis.

### DNA and RNA sample collection and extraction and metagenome and metatranscriptome sequencing.

Eleven mixed liquor samples were collected for DNA and RNA extraction across a cycle study consisting of a 30-min nonreaction phase devoid of ammonium and nitrite and a 70-min reaction phase with ammonium and nitrite adjusted to initial concentrations of 25 mg N/liter and 33 mg N/liter, respectively. Samples were collected every 10 min, snap-frozen in liquid nitrogen, and stored at −80°C. Briefly, DNA and RNA were purified in parallel using a ZR-Duet DNA/RNA miniprep kit (Zymo Research, USA) and a ZR soil and fecal RNA microprep kit (Zymo Research, USA), optimized for nucleic acid extraction from activated sludge. Following genomic DNA purification, a sequencing library was prepared using a modified version of the Illumina TruSeq DNA sample preparation protocol, with sequencing by Illumina HiSeq2500 with a 250-bp paired–end read length. rRNA was removed from purified total RNA by a subtractive hybridization–bead capture protocol using a Ribo-Zero Gold rRNA removal kit (epidemiology) (Illumina). Depleted RNA was cleaned with an RNA Clean & Concentrator kit 5 (Zymo Research) and Zymo-Spin IC columns (Zymo Research, USA), according to the manufacturer’s protocol. The RNA depletion output was quantified with a Qubit 2.0 fluorometer (Thermo Fisher Scientific) and a Qubit double-stranded RNA (dsRNA) high sensitivity assay (Thermo Fisher Scientific). Depleted RNA was analyzed immediately. cDNA was synthesized with a NEBNext RNA first-strand synthesis module (NEB) and NEBNext Ultra Directional RNA second-strand synthesis modules (NEB), according to the manufacturer’s recommendations. Double-stranded cDNA was introduced into the Illumina TruSeq stranded mRNA protocol at the “adenylate 3′ ends” step. Following standard quality control (QC) procedures, each library was uniquely tagged with an Illumina TruSeq LT RNA barcode to enable library pooling for sequencing.

### Metagenome and comparative genome analysis and transcriptome data analysis.

The raw gDNA FASTQ files were processed using cutadapt (version 1.16) with Python 3.6.3 in paired-end mode (with default arguments except —overlap 10 -m 30 -q 20,20). Single-sample assemblies were performed on the processed reads from each sample with SPAdes-3.12.0 (default parameters except —meta -k 21,33,55,77,99,127 -t 30), with open reading frames (ORFs) predicted from contigs of ≥500 bp using PRODIGAL v2.6.3 (default parameters except -f gff -p meta), and annotated against NCBI-NR (April 2018) using methods described by Law et al. (35). Anammox genomes were recovered using custom contig binning procedures developed in the R statistical computing environment. Following the work of Liu et al. (36), we used sequence similarity networks, obtained from BLASTP analysis of translated ORFs in our Anammox bin, and selected Anammox reference genomes to isolate genes of interest. Raw cDNA FASTQ files were processed using cutadapt (version 1.14) with Python 3.6.3 in paired end mode (with default arguments except —overlap 10 -m 30 -q 20,20). The processed reads were mapped to ORFs predicted from contigs using bowtie2-2.3.4.1. (default parameters except -q -t -p 30). The number of reads mapped to each ORF was counted using samtools-1.6 (idxstats option) and using reads per kilobase of transcript per million mapped reads (RPKM), calculated as a primary measure of gene expression.

### Microscopy and ThT staining.

Microscopic imaging was conducted with a Leica SPP8WLL confocal microscope using a 40× objective. Briefly, cryosectioned anammox biofilm (Leica Cryostats CM1950) (5 μm) gel formed during dialysis and purified protein were stained with 0.5% (wt/vol) ThT, each prepared using 0.1 N filtered HCl, for 15 min before being viewed under the microscope. ThT was excited at 405 nm and detected at 488 to 520 nm.

### Circular dichroism.

The anammox biofilm was resuspended in double-distilled water to achieve a UV absorbance reading of 1.0. Double-distilled water served as a blank. The samples were analyzed with a JASCO-815 spectropolarimeter in a 1-cm-path-length quartz cuvette containing a solution volume of 500 μl. An average of three scans was taken, and the buffer spectra were subtracted.

### Recombinant-protein expression.

Brosi_A1236 (GenBank accession no. GAN32721) cDNA was supplied by GenScript (Piscataway, NJ, USA). Sequences encoding amino acids 1254 to 1338 (construct 1), 1354 to 1439 (construct 2), and 1448 to 1551 (construct 3) were amplified by PCR and cloned into pNIC28-Bsa4 via ligation-independent cloning (37). The forward primer sequence was TACTTCCAATCCATGATTGTTACCCCGAGCCCGG, and the reverse primer sequence was TATCCACCTTTACTGTCAGGTAACGGTCACGGTCTTG. The verified plasmid was transformed into Escherichia coli BL21(DE3) Rosetta for protein expression.

Terrific Broth (750 ml) was inoculated with 20 ml E. coli BL21 seed cultures grown overnight. Cultures were incubated at 37°C in the LEX system (Harbinger Biotech) with aeration and agitation by the bubbling of filtered air. The LEX system temperature was reduced to 18°C when the culture optical density at 600 nm (OD_600_) reached 2.0, and the cultures were induced after 60 min with 0.5 mM IPTG (isopropyl-β-d-thiogalactopyranoside). Protein expression was allowed to continue overnight. Cells were harvested by centrifugation at 4,000 × *g* and 15°C for 10 min. The supernatants were discarded, and the cell pellets were resuspended in lysis buffer (1.5 ml/g cell pellet). The cell suspensions were stored at −80°C before purification.

Resuspended cell pellets were thawed and sonicated (Sonics Vibra-cell) at 70% amplitude. The lysate was clarified by centrifugation at 47,000 × *g* and 4°C for 25 min. The supernatants were filtered through 1.2-μm syringe filters and loaded onto an ÄKTAxpress system (GE Healthcare). The lysates were loaded on IMAC (immobilized metal affinity chromatography) columns. The columns were washed with 20 ml of 20 mM HEPES (pH 7.5), 300 mM NaCl, 10% (vol/vol) glycerol, 2 mM TCEP [Tris(2-carboxyethyl)phosphine hydrochloride]. The eluted proteins were collected and stored in sample loops on the system and injected into HiLoad 16/60 Superdex 75 prep-grade columns (GE Healthcare). Elution peaks were collected in 2-ml fractions and analyzed by SDS-PAGE. The entire purification was performed at 4°C. Relevant peaks were pooled, and TCEP was added to a total concentration of 2 mM. The protein sample was concentrated in Vivaspin 20 filter concentrators (VivaScience) at 15°C to approximately 15 mg/ml (<18 kDa, 5,000 MWCO; 19 to 49 kDa, 10,00 MWCO; >50 kDa, 30,000 MWCO). The final protein concentration was assessed by measuring absorbance at 280 nm on a NanoDrop ND-1000 spectrophotometer (NanoDrop Technologies). The final protein purity was assessed by SDS-PAGE. The final protein batch was then aliquoted into smaller fractions, frozen in liquid nitrogen, and stored at −80°C.

### Protein binding assay (microsphere).

Proteins were attached to carboxylated fluorescent microspheres (Fluoresbrite; yellow-green [YG] and polychromatic red) according to a modified protocol (38). Briefly, microspheres were washed twice by diluting 10 times with activation buffer (50 mM morpholineethanesulfonic acid [MES], pH 6.0) and centrifuged at 12,000 rpm for 5 min. After washing, microspheres were activated with 1-ethyl-3-[3-dimethylaminopropyl] carbodiimide (EDC) and *N*-hydroxysuccinimide (NHS) for 30 min and then washed twice with activation buffer. Protein constructs were used to coat the different-colored microspheres by incubation at 22°C for 2 h on an orbital shaker at 300 rpm. After incubation, coated microspheres were centrifuged to remove the excess proteins and brought to the original volume with activation buffer. Sonication was conducted between steps to ensure complete dispersal of the microspheres. Beads with different protein constructs were coupled, and 5 mM Ca^2+^ was added to the mixture. The mixtures were incubated for 1 h at 22°C under the shaking conditions described above. Fluorescence imaging was obtained using a Leica SP8 confocal laser electronic microscope. Microspheres coated with cadherin in the presence of CaCl_2_ were used as positive controls, whereas microspheres coated with bovine serum albumin were used as negative controls.

### Liquid-liquid condensation assay.

Liquid droplets were induced by incubating a Brosi_A1236 protein construct (amino acids 1355 to 1439) in 20% (wt/vol) PEG 8000 (polyethylene glycol; Sigma-Aldrich) and 20 mM Tris–125 mM NaCl–2 mM dithiothreitol (DTT) buffer to a final concentration of 100 μM. The mixture was incubated overnight at 4°C followed by imaging on Axio Observer.Z1 inverted wide-field microscope (Zeiss) with ×63 magnification.

Briefly, the protein isolate was labeled with Alexa Fluor 647 by adding 50 μl of purified protein at a concentration of 2.5 mg/ml to 25 μl of the reactive dye (Alexa Fluor 647 protein labeling kit; Thermo Fisher Scientific), which was resuspended in 1× phosphate-buffered saline (PBS) and 50 μl of 1 M sodium bicarbonate solution, pH 8.3. The reaction mixture was stirred at room temperature for 1 h. Separation of the Alexa Fluor 647-tagged protein was then done by passing the reaction mixture through the purification resin provided in the kit. The first eluted colored band was collected as the Alexa Fluor 647-tagged protein.

Alexa Fluor 647-tagged purified protein was concentrated using Amicon Ultra 0.5-ml centrifugal filters (Merck) to give 1.6 mg/ml of fluorescently tagged protein. Liquid droplets of the Alexa Fluor 647-tagged protein were induced by incubating 5 μl of 40% PEG 8000 with 4 μl of sample and 1 μl of 20 mM Tris–125 mM NaCl–2 mM DTT at 4°C for 30 min. The sample was then imaged on an Axio Observer.Z1 inverted wide-field microscope (Zeiss) with ×40 magnification.

### Cell wetting assay (i.e., in liquid droplets).

Briefly, Brosi_A1236 liquid droplets were induced using the protocol described above. Carboxylate-modified latex microspheres (0.5 μm; yellow-green) were first washed three times with 50 mM MES buffer, pH 6.0. The microspheres were sonicated and centrifuged at 14,000 × *g* during every wash. Microspheres were resuspended in 50 mM MES buffer at a concentration of 10% (wt/vol) before being mixed with liquid droplets.

Ten milliliters of an overnight culture of green fluorescent protein (GFP)-tagged Pseudomonas aeruginosa PAO1 wild type (WT) was incubated in lysogeny broth (LB) at 37°C with shaking.

Coalescence of microspheres and GFP-tagged PAO1 WT cells to liquid droplets was observed using an Axio Observer.Z1 inverted wide-field microscope (Zeiss) with ×40 magnification. Liquid droplet solutions were prepared, and microspheres and PAO1 WT cell culture were added to final concentrations of 1% (wt/vol) and an optical density of 0.14, respectively.

### Rheology measurements.

A Haake Mars 60 (Thermo Scientific) stress-controlled rotational rheometer, with a Peltier controlled element at 25°C, was used for rheological measurements. A 35-mm-diameter parallel plate geometry was used with smooth titanium plates to measure storage and loss moduli and complex viscosity. One hundred fifty microliters of the sample was deposited on the plates. The plates were closed to a gap of 150 μm. Small and reversible oscillations with frequency (ω) varying from 0.1 to 10/s were applied with a fixed strain of 0.01, as this was found to be within the linear visco-elastic region of deformation.

### Transmission electron microscopy by negative staining.

Brosi_A1236 in 20 mM HEPES (pH 8.0) was mixed with 0.1 M HEPES (pH 7.4) with or without CaCl_2_ (final protein concentration, 0.1 mg/ml; final CaCl_2_ concentration, 10 mM) for 60 min at 22°C. Samples were applied to freshly glow-discharged carbon-coated grids (400 mesh copper; Ted Pella, Inc., CA, USA), fixed with 2% glutaraldehyde (Electron Microscopy Sciences [EMS], PA, USA), rinsed in double-distilled H_2_O, negatively stained with 4% uranyl acetate (EMS), and air-dried. Samples were analyzed with a JEM-2200FS transmission electron microscope (JEOL Ltd., Japan) operated at 200 kV. Images were acquired using a TVIPS TemCam-F416 complementary metal oxide semiconductor (CMOS) camera (16 bit, 4K resolution). TEM diffraction patterns were recorded using selected area diffraction (50-μm aperture).

### Data availability.

Raw metatranscriptomic data used in this study is available from NCBI BioProject ID PRJNA658207.

## References

[1] Flemming HC, Wingender J, Szewzyk U, Steinberg P, Rice SA, Kjelleberg S 2016 Biofilms: an emergent form of bacterial life. Nat Rev Microbiol 14:563–575. doi:10.1038/nrmicro.2016.94. 27510863

[2] Seviour T, Derlon N, Dueholm MS, Flemming H-C, Girbal-Neuhauser E, Horn H, Kjelleberg S, van Loosdrecht MCM, Lotti T, Malpei MF, Nerenberg R, Neu TR, Paul E, Yu H, Lin Y 2019 Extracellular polymeric substances of biofilms: suffering from an identity crisis. Water Res 151:1–7. doi:10.1016/j.watres.2018.11.020. 30557778

[3] Strous M, Heijnen JJ, Kuenen JG, Jetten MSM 1998 The sequencing batch reactor as a powerful tool for the study of slowly growing anaerobic ammonium-oxidizing microorganisms. Appl Microbiol Biotechnol 50:589–596. doi:10.1007/s002530051340.

[4] Kuenen JG 2008 Anammox bacteria: from discovery to application. Nat Rev Microbiol 6:320–326. doi:10.1038/nrmicro1857. 18340342

[5] Sonthiphand P, Hall MW, Neufeld JD 2014 Biogeography of anaerobic ammonia-oxidizing (anammox) bacteria. Front Microbiol 5:399–399. doi:10.3389/fmicb.2014.00399. 25147546 PMC4123730

[6] Kindaichi T, Tsushima I, Ogasawara Y, Shimokawa M, Ozaki N, Satoh H, Okabe S 2007 *In situ* activity and spatial organization of anaerobic ammonium-oxidizing (anammox) bacteria in biofilms. Appl Environ Microbiol 73:4931–4939. doi:10.1128/AEM.00156-07. 17526785 PMC1951037

[7] Xiao Y, Zeng GM, Yang ZH, Liu Y, Ma YH, Yang L, Wang RJ, Xu ZY 2009 Coexistence of nitrifiers, denitrifiers and anammox bacteria in a sequencing batch biofilm reactor as revealed by PCR-DGGE. J Appl Microbiol 106:496–505. doi:10.1111/j.1365-2672.2008.04017.x. 19200316

[8] Lotti T, Carretti E, Berti D, Martina MR, Lubello C, Malpei F 2019 Extraction, recovery and characterization of structural extracellular polymeric substances from anammox granular sludge. J Environ Manage 236:649–656. doi:10.1016/j.jenvman.2019.01.054. 30772722

[9] Boleij M, Pabst M, Neu TR, van Loosdrecht MCM, Lin Y 2018 Identification of glycoproteins isolated from extracellular polymeric substances of full-scale anammox granular sludge. Environ Sci Technol 52:13127–13135. doi:10.1021/acs.est.8b03180. 30335377 PMC6256349

[10] Lotti T, Carretti E, Berti D, Montis C, Del Buffa S, Lubello C, Feng C, Malpei F 2019 Hydrogels formed by anammox extracellular polymeric substances: structural and mechanical insights. Sci Rep 9:11633. doi:10.1038/s41598-019-47987-8. 31406144 PMC6690907

[11] Boleij M, Seviour T, Wong LL, van Loosdrecht MCM, Lin Y 2019 Solubilization and characterization of extracellular proteins from anammox granular sludge. Water Res 164:114952. doi:10.1016/j.watres.2019.114952. 31408759

[12] Wong LL, Natarajan G, Boleij M, Thi SS, Winnerdy FR, Mugunthan S, Lu Y, Lee J-M, Lin Y, van Loosdrecht M, Law Y, Kjelleberg S, Seviour T 2020 Extracellular protein isolation from the matrix of anammox biofilm using ionic liquid extraction. Appl Microbiol Biotechnol 104:3643–3654. doi:10.1007/s00253-020-10465-7. 32095864

[13] van Teeseling MCF, de Almeida NM, Klingl A, Speth DR, Op den Camp HJM, Rachel R, Jetten MSM, van Niftrik L 2014 A new addition to the cell plan of anammox bacteria: “*Candidatus* Kuenenia stuttgartiensis” has a protein surface layer as the outermost layer of the cell. J Bacteriol 196:80–89. doi:10.1128/JB.00988-13. 24142254 PMC3911120

[14] Yoshizawa T, Nozawa R-S, Jia TZ, Saio T, Mori E 2020 Biological phase separation: cell biology meets biophysics. Biophys Rev 12:519–539. doi:10.1007/s12551-020-00680-x. 32189162 PMC7242575

[15] Bergeron-Sandoval L-P, Safaee N, Michnick Stephen W 2016 Mechanisms and consequences of macromolecular phase separation. Cell 165:1067–1079. doi:10.1016/j.cell.2016.05.026. 27203111

[16] Shin Y, Brangwynne CP 2017 Liquid phase condensation in cell physiology and disease. Science 357:eaaf4382. doi:10.1126/science.aaf4382. 28935776

[17] Faltova L, Küffner AM, Hondele M, Weis K, Arosio P 2018 Multifunctional protein materials and microreactors using low complexity domains as molecular adhesives. ACS Nano 12:9991–9999. doi:10.1021/acsnano.8b04304. 30216718

[18] Seviour T, Yuan Z, van Loosdrecht MCM, Lin Y 2012 Aerobic sludge granulation: a tale of two polysaccharides? Water Res 46:4803–4813. doi:10.1016/j.watres.2012.06.018. 22776210

[19] Yachdav G, Kloppmann E, Kajan L, Hecht M, Goldberg T, Hamp T, Hönigschmid P, Schafferhans A, Roos M, Bernhofer M, Richter L, Ashkenazy H, Punta M, Schlessinger A, Bromberg Y, Schneider R, Vriend G, Sander C, Ben-Tal N, Rost B 2014 PredictProtein—an open resource for online prediction of protein structural and functional features. Nucleic Acids Res 42:W337–W343. doi:10.1093/nar/gku366. 24799431 PMC4086098

[20] Yang J, Zhang Y 2015 Protein structure and function prediction using I-TASSER. Curr Protoc Bioinformatics 52:5.8.1–5.8.15. 26678386 10.1002/0471250953.bi0508s52 PMC4871818

[21] Micsonai A, Wien F, Bulyáki É, Kun J, Moussong É, Lee Y-H, Goto Y, Réfrégiers M, Kardos J 2018 BeStSel: a web server for accurate protein secondary structure prediction and fold recognition from the circular dichroism spectra. Nucleic Acids Res 46:W315–W322. doi:10.1093/nar/gky497. 29893907 PMC6031044

[22] Groenning M, Olsen L, van de Weert M, Flink JM, Frokjaer S, Jørgensen FS 2007 Study on the binding of thioflavin T to β-sheet-rich and non-β-sheet cavities. J Struct Biol 158:358–369. doi:10.1016/j.jsb.2006.12.010. 17289401

[23] Dueholm MS, Søndergaard MT, Nilsson M, Christiansen G, Stensballe A, Overgaard MT, Givskov M, Tolker-Nielsen T, Otzen DE, Nielsen PH 2013 Expression of Fap amyloids in *Pseudomonas aeruginosa*, *P. fluorescens*, and *P. putida* results in aggregation and increased biofilm formation. Microbiologyopen 2:365–382. doi:10.1002/mbo3.81. 23504942 PMC3684753

[24] Gottenbos B, Grijpma DW, van der Mei HC, Feijen J, Busscher HJ 2001 Antimicrobial effects of positively charged surfaces on adhering Gram-positive and Gram-negative bacteria. J Antimicrob Chemother 48:7–13. doi:10.1093/jac/48.1.7. 11418507

[25] An P, Xu X, Yang F, Li Z 2013 Comparison of the characteristics of anammox granules of different sizes. Biotechnol Bioproc E 18:446–454. doi:10.1007/s12257-012-0728-4.

[26] Tompa P 2009 Structural disorder in amyloid fibrils: its implication in dynamic interactions of proteins. FEBS J 276:5406–5415. doi:10.1111/j.1742-4658.2009.07250.x. 19712107

[27] Dueholm MS, Nielsen SB, Hein KL, Nissen P, Chapman M, Christiansen G, Nielsen PH, Otzen DE 2011 Fibrillation of the major curli subunit CsgA under a wide range of conditions implies a robust design of aggregation. Biochemistry 50:8281–8290. doi:10.1021/bi200967c. 21877724 PMC3724407

[28] Seviour T, Pijuan M, Nicholson T, Keller J, Yuan Z 2009 Gel-forming exopolysaccharides explain basic differences between structures of aerobic sludge granules and floccular sludges. Water Res 43:4469–4478. doi:10.1016/j.watres.2009.07.018. 19682721

[29] Alberti S 2017 The wisdom of crowds: regulating cell function through condensed states of living matter. J Cell Sci 130:2789–2796. doi:10.1242/jcs.200295. 28808090

[30] Chung S, Shin S-H, Bertozzi CR, De Yoreo JJ 2010 Self-catalyzed growth of S layers via an amorphous-to-crystalline transition limited by folding kinetics. Proc Natl Acad Sci U S A 107:16536–16541. doi:10.1073/pnas.1008280107. 20823255 PMC2944705

[31] Darling AL, Uversky VN 2018 Intrinsic disorder and posttranslational modifications: the darker side of the biological dark matter. Front Genet 9:158. doi:10.3389/fgene.2018.00158. 29780404 PMC5945825

[32] de Graaff MS, van den Brand T, Roest K, Zandvoort M, Duin O, van Loosdrecht M 2016 Full-scale highly-loaded wastewater treatment processes (A-stage) to increase energy production from wastewater: performance and design guidelines. Environ Eng Sci 33:571–577. doi:10.1089/ees.2016.0022.

[33] Lu Y, Natarajan G, Nguyen TQN, Thi SS, Arumugam K, Seviour TW, Williams RBH, Wuertz S, Law Y 2020 Species level enrichment of AnAOB and associated growth morphology under the effect of key metabolites. bioRxiv https://www.biorxiv.org/content/biorxiv/early/2020/02/05/2020.02.04.934877.full.pdf.

[34] Seviour T, Weerachanchai P, Roizman D, Hinks J, Bai L, Rice S, Lee J-M, Kjelleberg S 2015 Solvent optimization for bacterial extracellular matrices: a solution for the insoluble. RSC Adv 5:7469–7478. doi:10.1039/C4RA10930A.

[35] Law Y, Kirkegaard RH, Cokro AA, Liu X, Arumugam K, Xie C, Stokholm-Bjerregaard M, Drautz-Moses DI, Nielsen PH, Wuertz S, Williams RBH 2016 Integrative microbial community analysis reveals full-scale enhanced biological phosphorus removal under tropical conditions. Sci Rep 6:25719. doi:10.1038/srep25719. 27193869 PMC4872125

[36] Liu X, Arumugam K, Natarajan G, Seviour TW, Drautz-Moses DI, Wuertz S, Law Y, Williams RBH 2018 Draft genome sequence of a “*Candidatus* Brocadia” bacterium enriched from activated sludge collected in a tropical climate. Genome Announc 6:e00406-18. doi:10.1128/genomeA.00406-18. 29748410 PMC5946052

[37] Savitsky P, Bray J, Cooper CD, Marsden BD, Mahajan P, Burgess-Brown NA, Gileadi O 2010 High-throughput production of human proteins for crystallization: the SGC experience. J Struct Biol 172:3–13. doi:10.1016/j.jsb.2010.06.008. 20541610 PMC2938586

[38] Poetz O, Luckert K, Herget T, Joos TO 2009 Microsphere-based co-immunoprecipitation in multiplex. Anal Biochem 395:244–248. doi:10.1016/j.ab.2009.08.002. 19665442

