# Supplementary material for: Phase Transitions by an Abundant Protein in the Anammox Extracellular Matrix Mediate Cell-to-Cell Aggregation and Biofilm Formation
Source: mBio. 2020 Sep 8;11(5):e02052-20. doi: 10.1128/mBio.02052-20 (PMC7482068; doi:10.1128/mBio.02052-20)
Supplement: TEXT S1 [file mBio.02052-20-s0001.docx]

**Materials and Methods**

***Pseudomonas aeruginosa* biofilm growth assay (i.e. ThT control)**

Ten milliliter aliquots of planktonic pre-culture of *P. aeruginosa* PAO1 (LB, 200 rpm, 37°C, OD_600_ 2.40, 16 h) were diluted 50 times with LB in 2 L Erlenmeyer flasks and incubated for 4 d under static conditions. The cultures were centrifuged at 10,000 x *g* for 15 min, the supernatant removed by decanting, and the biofilm then collected for staining and microscopic analysis. 20 μL of the biofilm were deposited onto a glass slide. 100 μL of Thioflavin T (0.5% w/v) and 200 μL of FilmTracer™️ SYPRO™️ Ruby biofilm matrix stain were then added simultaneously to cover the surface of the biofilm, followed by incubation at room temperature for 30 minutes. The extracellular matrix-specific FilmTracer™️ SYPRO™️ Ruby biofilm matrix stain was used to demonstrate that ThT does not stain the the extracellular matrix of *P. aeruginosa* (1). The Ruby matrix stain was excited at 450 nm and detected at 610 nm, and ThT was excited at 405 nm and detected at 488-520 nm.

1. Srikantha T, Daniels KJ, Pujol C, Kim E, Soll DR. 2013. Identification of genes upregulated by the transcription factor Bcr1 that are involved in impermeability, impenetrability, and drug resistance of Candida albicans a/α biofilms. *Eukaryot Cell* **12**:875-88.
