# Supplementary material for: Phase Transitions by an Abundant Protein in the Anammox Extracellular Matrix Mediate Cell-to-Cell Aggregation and Biofilm Formation
Source: mBio. 2020 Sep 8;11(5):e02052-20. doi: 10.1128/mBio.02052-20 (PMC7482068; doi:10.1128/mBio.02052-20)
Supplement: FIG S1 [file mBio.02052-20-sf001.pdf]

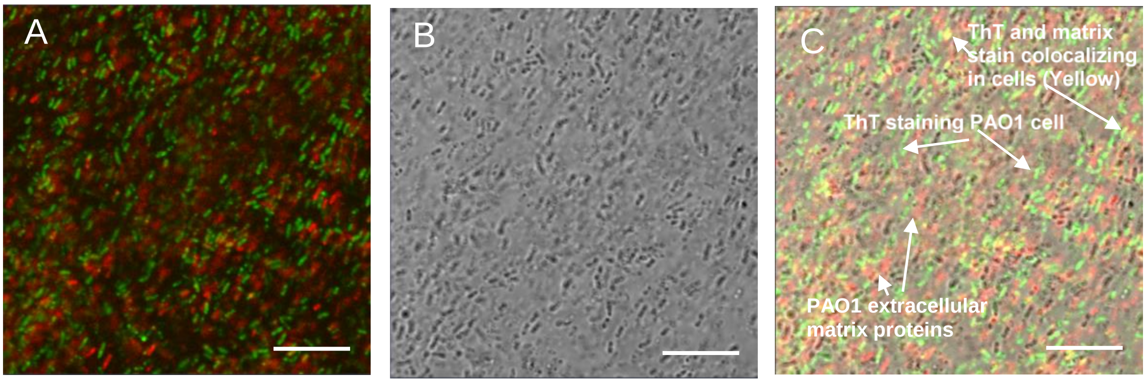

Figure S1: (A) Confocal (Green: Thioflavin T, 0.5% w/v; Red: FilmTracer™ SYPRO™ Ruby biofilm matrix stain) (B) Brightfield and (C) overlapping micrographs of 4-day old *Pseudomonas aeruginosa* (PAO1) biofilm. These show that coincident binding to Thioflavin T and Ruby takes place within cells only (i.e. yellow-colored) and not within extracellular matrix; and that only the extracellular region is stained by Ruby biofilm matrix stain (i.e. red regions between cells). Scale bar represents 10 µm.
