# Supplementary material for: Phase Transitions by an Abundant Protein in the Anammox Extracellular Matrix Mediate Cell-to-Cell Aggregation and Biofilm Formation
Source: mBio. 2020 Sep 8;11(5):e02052-20. doi: 10.1128/mBio.02052-20 (PMC7482068; doi:10.1128/mBio.02052-20)
Supplement: FIG S2 [file mBio.02052-20-sf002.pdf]

A

i)
VTPTPGVTPTPTGVGRILGQVTDSISGAGINGAAVSLDTGEQTVTSTVGGQDGVYSFDNVAVGDRTITAAATGYITN
TKTVTVTDGGVADGRIE

ii)
LTPEVIVTPSPVASPTP**PPQVGRI**LGQVTDSETSAGINGATVSLDTGEQTVTSTVG**GQD**GVYSFNDVAVGDRTITAA
ATGYITNTKTVTVTDGGVADGRIE

B

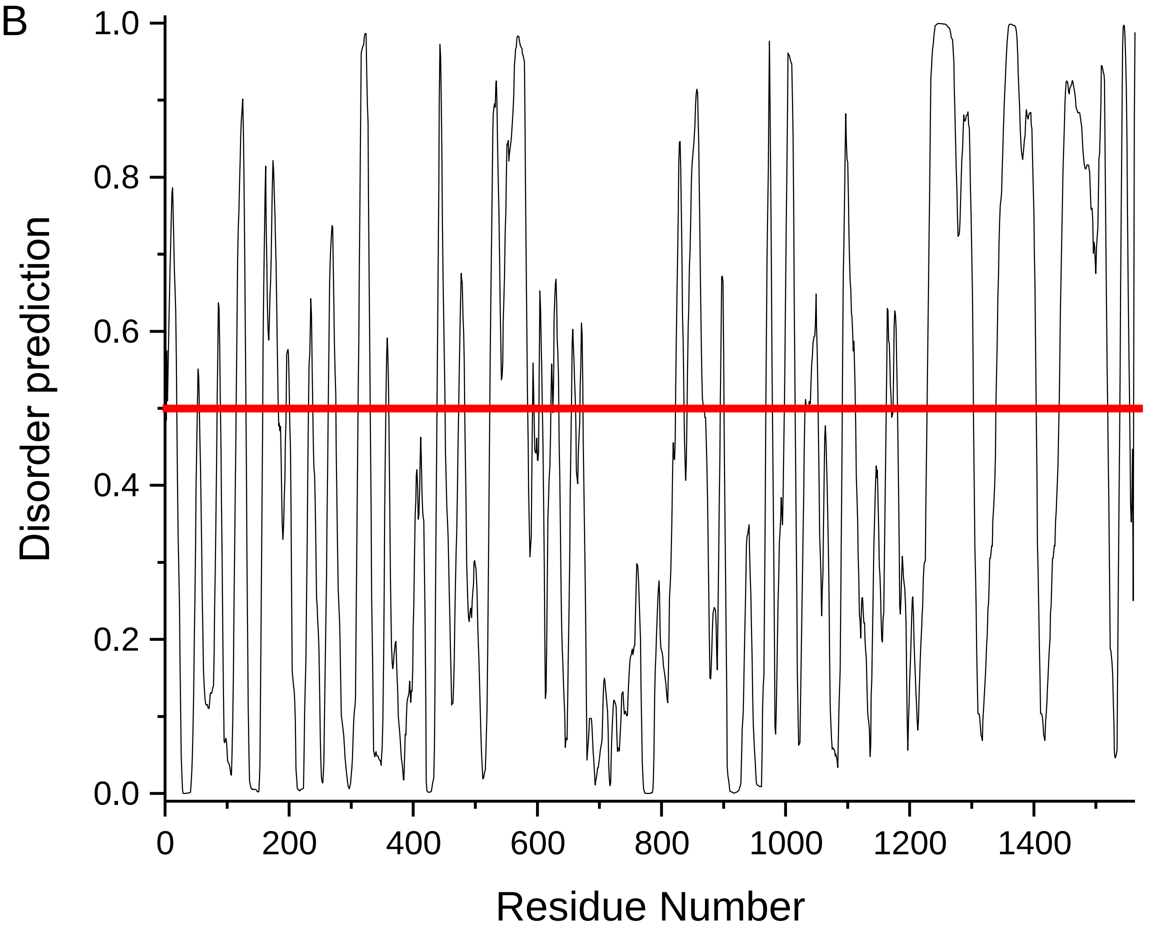

Figure S2: A) C-terminal repeat domains of Brosi_A1236 from amino acids i) 1254-1338 and ii) 1354-1439. B) Intrinsic disorder prediction for Brosi_A1236 by Predictor of Natural Disordered Regions (PONDR), with disorder prediction score > 0.5 indicating disordered regions.
