# Supplementary material for: Phase Transitions by an Abundant Protein in the Anammox Extracellular Matrix Mediate Cell-to-Cell Aggregation and Biofilm Formation
Source: mBio. 2020 Sep 8;11(5):e02052-20. doi: 10.1128/mBio.02052-20 (PMC7482068; doi:10.1128/mBio.02052-20)
Supplement: FIG S3 [file mBio.02052-20-sf003.pdf]

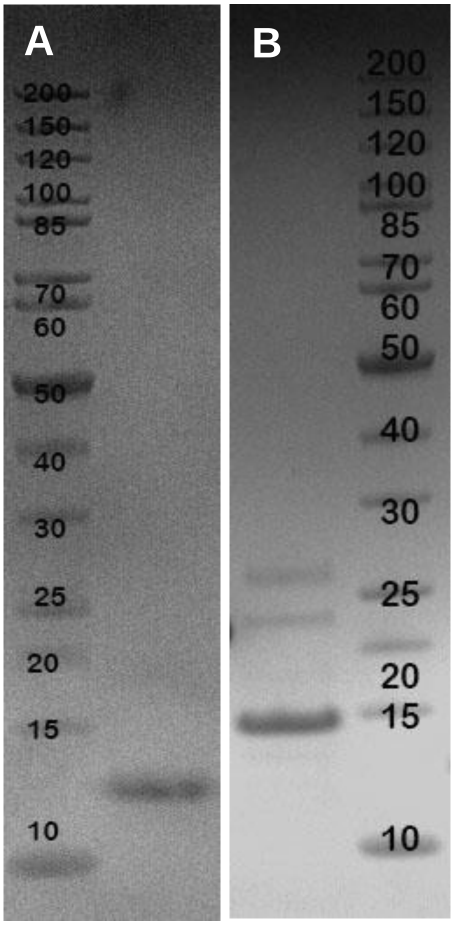

Figure S3: Coomassie stain of SDS-page gel of anammox biofilm surface protein construct A) repeat domain 1 (i.e. amino acids 1254-1338) and B) repeat domain 2 (i.e. amino acids 1354-1439) recombinantly expressed with *Escherichia coli.*
