# Supplementary material for: Phase Transitions by an Abundant Protein in the Anammox Extracellular Matrix Mediate Cell-to-Cell Aggregation and Biofilm Formation
Source: mBio. 2020 Sep 8;11(5):e02052-20. doi: 10.1128/mBio.02052-20 (PMC7482068; doi:10.1128/mBio.02052-20)
Supplement: FIG S4 [file mBio.02052-20-sf004.pdf]

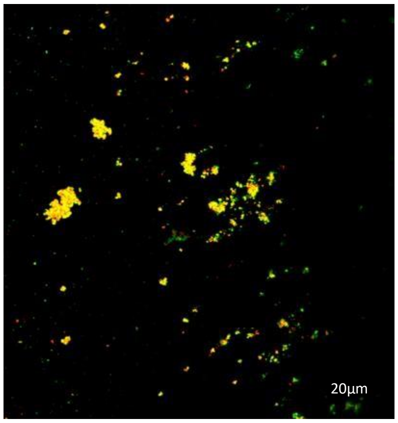

Figure S4: Protein binding assay showing the coalescence of green and red microspheres with both pre-incubated with cadherin in the presence of 20 mM calcium (i.e. positive control)
