# Supplementary material for: Phase Transitions by an Abundant Protein in the Anammox Extracellular Matrix Mediate Cell-to-Cell Aggregation and Biofilm Formation
Source: mBio. 2020 Sep 8;11(5):e02052-20. doi: 10.1128/mBio.02052-20 (PMC7482068; doi:10.1128/mBio.02052-20)
Supplement: FIG S5 [file mBio.02052-20-sf005.pdf]

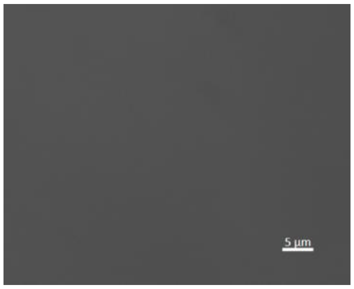

Figure S5: Brightfield micrograph of aqueous solution of 20 mM Tris, pH 7.5, 125 mM NaCl, 2 mM DTT, 4 °C, following the addition of PEG (20% (w/v))
